# A novel intramuscular Interstitial Cell of Cajal is a candidate for generating pacemaker activity in the mouse internal anal sphincter

**DOI:** 10.1038/s41598-020-67142-y

**Published:** 2020-06-25

**Authors:** Karen I. Hannigan, Aaron P. Bossey, Holly J. L. Foulkes, Bernard T. Drumm, Salah A. Baker, Sean M. Ward, Kenton M. Sanders, Kathleen D. Keef, Caroline A. Cobine

**Affiliations:** Department of Physiology and Cell Biology, University of Nevada, Reno School of Medicine, Reno, NV 89557 USA

**Keywords:** Physiology, Gastroenterology, Gastrointestinal system

## Abstract

The internal anal sphincter (IAS) generates phasic contractions and tone. Slow waves (SWs) produced by interstitial cells of Cajal (ICC) underlie phasic contractions in other gastrointestinal regions. SWs are also present in the IAS where only intramuscular ICC (ICC-IM) are found, however the evidence linking ICC-IM to SWs is limited. This study examined the possible relationship between ICC-IM and SWs by recording Ca^2+^ transients in mice expressing a genetically-encoded Ca^2+^-indicator in ICC (Kit-Cre-GCaMP6f). A role for L-type Ca^2+^ channels (Cav_L_) and anoctamin 1 (ANO1) was tested since each is essential for SW and tone generation. Two distinct ICC-IM populations were identified. Type I cells (36% of total) displayed localised asynchronous Ca^2+^ transients not dependent on Cav_L_ or ANO1; properties typical of ICC-IM mediating neural responses in other gastrointestinal regions. A second novel sub-type, i.e., Type II cells (64% of total) generated rhythmic, global Ca^2+^ transients at the SW frequency that were synchronised with neighbouring Type II cells and were abolished following blockade of either Cav_L_ or ANO1. Thus, the spatiotemporal characteristics of Type II cells and their dependence upon Cav_L_ and ANO1 all suggest that these cells are viable candidates for the generation of SWs and tone in the IAS.

## Introduction

The internal anal sphincter (IAS) is responsible for approximately 70% of resting anal pressure; an important property for maintaining faecal continence^1,2^. Reports suggest that approximately 43% of faecal incontinence cases may be related to disturbances in IAS motility^3^. Unlike the majority of the gastrointestinal (GI) tract, the IAS spontaneously develops tone; a critical feature for raising pressure in the anal canal. While others have suggested that the IAS is a “purely tonic muscle” that develops tone as a result of enhanced myofilament sensitivity to Ca^2+^ ^4,5^, we have found that the IAS is fundamentally a phasic smooth muscle that generates tone as a result of the summation of phasic contractions^6–10^. The IAS exhibits slow wave (SW) activity, the electrophysiological events that give rise to phasic contractions in many regions of the GI tract^11^. SWs, phasic contractions and tone rely upon Ca^2+^ influx via voltage-gated L-type Ca^2+^ channels (Cav_L_)^8,12–14^. Thus, mechanisms regulating Ca^2+^ entry are clearly fundamental for tone development in the IAS.

Interstitial cells of Cajal (ICC) are present throughout the GI tract and studies of ICC in non-sphincter muscles indicate that SWs are generated by specialised ICC located predominantly at the myenteric (ICC-MY) and/or submucosal (ICC-SM) edges of the circular muscle layer^11^. Pacemaker ICC in these regions are coupled electrically to one another and to adjacent smooth muscle cells (SMCs) via gap junctions allowing conduction of SWs from ICC to SMCs where excitation-contraction coupling occurs^15^. These cells are typically highly-branched stellate-shaped cells^16,17^. In contrast, another population of spindle-shaped intramuscular ICC (ICC-IM) are involved in neuromuscular transmission^16,18^. ICC are also present in the IAS of various species but their distribution and morphology differs significantly from that of non-sphincteric muscles^19–23^. Importantly, the density of ICC-MY and ICC-SM declines from rectum to IAS with only ICC-IM present in the distal IAS^21,23^. SW amplitude and frequency are greatest in the distal IAS^8,23,24^ and thus we hypothesise that ICC-IM could be the pacemaker cells that generate SWs in the IAS.

IAS-SWs differ significantly from intestinal SWs. Intestinal SWs persist in the presence of antagonists of Cav_L_^25^, but IAS-SWs are inhibited by these antagonists, suggesting an essential role for Cav_L_ in these events in the IAS^7,8,14,26^. In addition, intestinal SWs have a more rapid upstroke due to the opening of T-type Ca^2+^ channels (Cav_T_) which activate and deactivate at a more negative membrane potential (Em) than Cav_L_^27,28^. The predominance of Cav_L_ as a mediator of SWs in the IAS is not surprising because “resting” Em in the IAS (−43 to −49 mV mouse IAS^7,23,26^) is more depolarised than intestine (−65 mV mouse jejunum^29^) and thus Cav_T_ channels would be substantially inactivated^7,8,30^.

SWs in the IAS and other GI muscles are dependent upon the Ca^2+^-activated Cl^-^ channel, Anoctamin-1 (ANO1, encoded in mice by *Ano1*). ANO1 is highly expressed in ICC throughout the GI tract including the IAS^14,29,31,32^, but this conductance is not resolved in SMCs or the other type of interstitial cell found in GI muscles, platelet-derived growth factor receptor alpha-positive (PDGFRα^+^) cells. The cell-specific expression of ANO1 is important because antagonists of this conductance or genetic deactivation of *Ano1* can be used to examine the functional role of ICC in intact muscles. ANO1 antagonists greatly reduce or abolish SWs^14,29^, and SWs fail to develop in ANO1 deficient mice^29,33–35^. Localised intracellular Ca^2+^ transients in ICC activate ANO1 channels, causing depolarisation, activation of voltage-gated Ca^2+^ channels and SW generation^29,31,36–38^. Since Cav_L_ and ANO1 antagonists block SWs as well as tone in the IAS we have proposed that IAS-SWs are important for tone generation^7,8,13,14,26^.

The current study utilised transgenic mice that express the genetically encoded Ca^2+^ indicator, GCaMP6f, in a cell-specific manner to visualise intracellular Ca^2+^ events in ICC-IM in the distal IAS *in situ*. The spatiotemporal properties of Ca^2+^ transients were measured in order to determine if ICC-IM generate pacemaker activity in the IAS. The effects of inhibition of Cav_L_, hyperpolarisation of the Em and blockade of ANO1, were examined as these characteristics have previously been shown to alter the electrical activity, phasic contractions and tone in the IAS.

## Results

### Two functionally distinct subtypes of ICC-IM are present in the IAS

**Figure 1:**
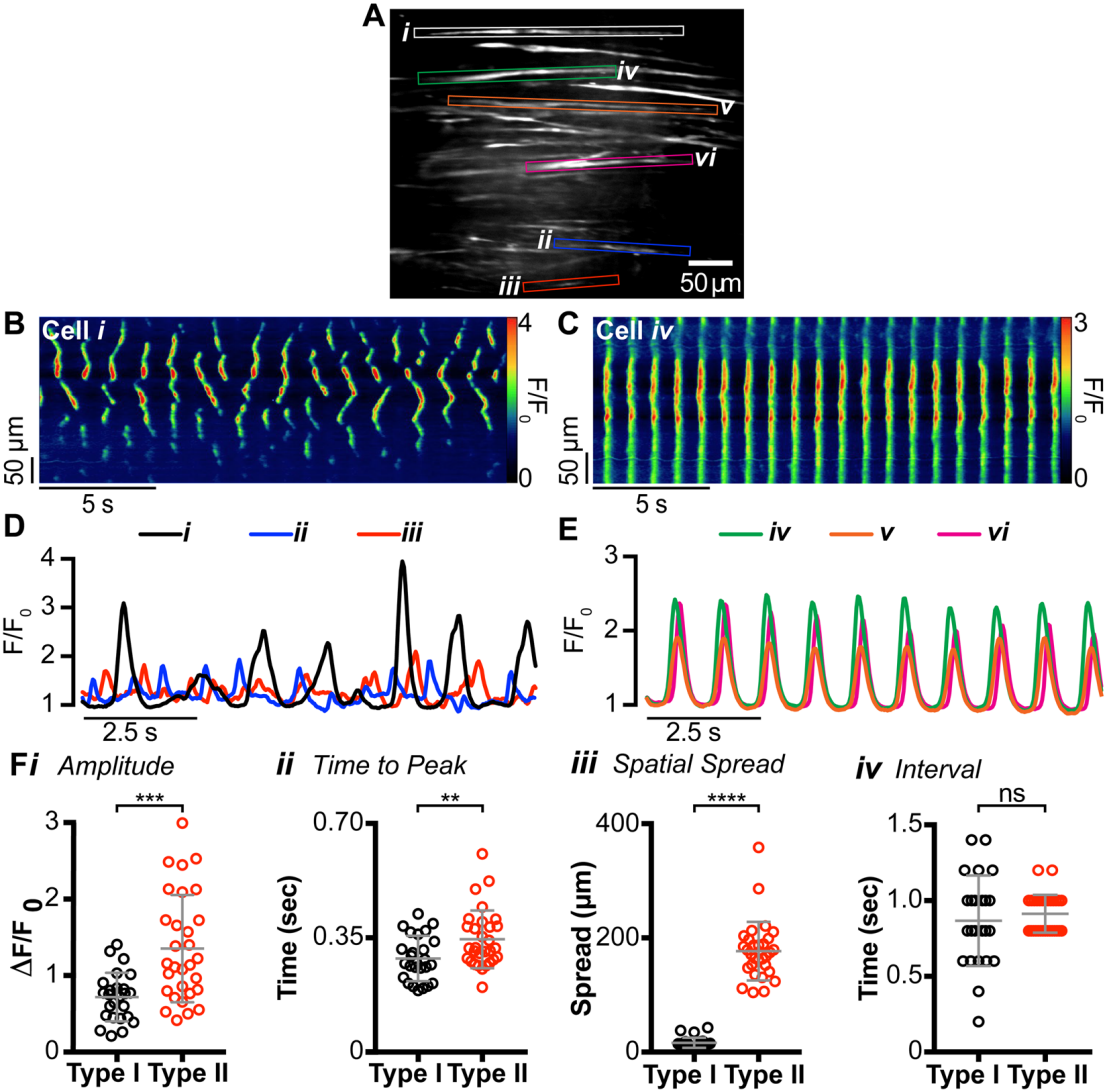
Two distinct populations of intramuscular interstitial cells of Cajal (ICC-IM) are present in the internal anal sphincter (IAS). **(A)** Frame of movie showing Ca^2+^ transients in two populations of GCaMP6f^+^ cells within the distal IAS (left, see also Supplemental Fig. S1). **(B**,**C)** Representative spatio-temporal (ST) maps created from cell *i* and *iv* comparing Ca^2+^ transients in Type I **(B)** and Type II cells **(C)**. **(D**,**E)** Superimposed plot profiles of Ca^2+^ activity in adjacent cells highlighted in A, demonstrating the asynchrony of Type I cells **(D)** and the synchrony of Type II cells **(E)**. **(F)** Scatter plots comparing **(*i*)** amplitude (*P* = 0.008), **(*ii*)** time to peak (*P* = 0.0057), **(*iii*)** spatial spread (*P* < 0.0001) and **(*iv*)** modal interval (*P* = 0.4413) of Ca^2+^ transients in Type I (○) and Type II ICC (○). Unpaired t test; Type I, n = 24, N = 15; Type II, n = 30, N = 18.

Ca^2+^ transients in ICC-IM in the distal IAS were imaged *in situ* using a spinning-disk confocal microscope. Initial experiments revealed two distinct patterns of Ca^2+^ transients in different spindle-shaped cell populations within the same field of view (FOV; Fig. 1A). Cell types were distinguished based upon differences in the characteristics of Ca^2+^ transients. Type I cells generated asynchronous Ca^2+^ transients that originated from multiple active sites and spread only short distances within the cell (Fig. 1A; cells *i*, *ii* and *iii* and 1B). In contrast, Type II cells generated synchronised, rhythmic Ca^2+^ transients that spread globally throughout the whole cell (Fig. 1A; cells *iv*, *v* and *vi* and 1C). By superimposing the plot profiles of Ca^2+^ transients from adjacent cells it is apparent that Type I cell activity was not synchronised within or between cells (Fig. 1B,D) whereas Type II cell activity was highly synchronised between neighbouring cells (Fig. 1C,E).

Detailed analysis from all recordings indicated that there was a significantly greater mean amplitude (*P* = 0.008), mean time to peak (*P* = 0.006), and mean spatial spread (*P* = 0.0001) of Ca^2+^ transients in Type II cells whereas the modal interval between Ca^2+^ transients was not significantly different (*P* = 0.441) between Type I and Type II cells (Fig. 1F). However, with regard to the modal interval between Ca^2+^ transients these were clustered tightly around the mean in Type II cells. This property is in keeping with their synchronised, rhythmic behaviour. In contrast, Type I cell intervals were distributed over a much wider range, commensurate with the generation of localised, asynchronous Ca^2+^ transients.

The rhythmic, highly synchronised behaviour of Type II cells makes it possible for these cells to be the pacemaker cells that generate IAS-SWs. To provide further insight into this relationship we determined the average frequency of Type II cell Ca^2+^ transients from the modal interval between Ca^2+^ transients. This analysis revealed an average Ca^2+^ transient frequency for Type II cells of 66.8 ± 1.6 cycles per min (cpm; n = 30, N = 18); a value that is well within the range reported for SWs at the distal end of the IAS (69.7 ± 3.2 cpm^23^). These data are consistent with the hypothesis that a relationship exists between Ca^2+^ transients in ICC-IM and SWs in the IAS.

### Distribution of Type I and Type II cells in the rectoanal region

**Figure 2:**
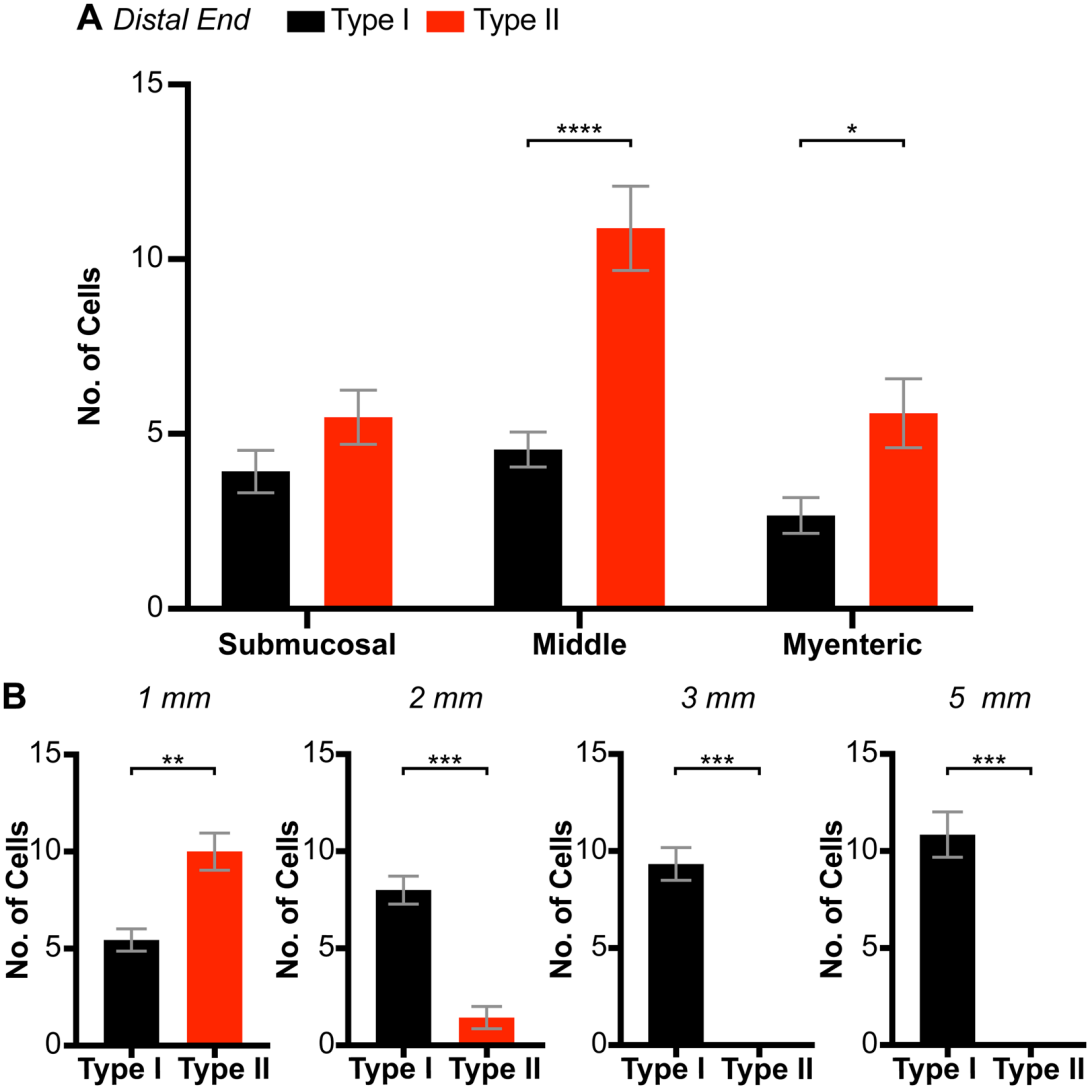
Distribution of Type I and Type II GCaMP6f^+^ cells in the internal anal sphincter (IAS) and rectum. **(A)** Bar graphs showing the distribution of Type I (black) and Type II cells (red) within the submucosal third, middle third and myenteric third of the circular muscle layer at the distal end of the IAS. *P = 0.034, ****P = 0.0001, N = 27, Two-way ANOVA with Šidák *post-hoc* test. **(B)** Distribution of Type I and Type II cells in the middle third of the muscle layer at increasing distance from the distal end of the IAS. Paired t test; 1 mm: **P = 0.0086, N = 7, 2 mm: ***P = 0.0007, N = 7, 3 mm: ***P = 0.0001, N = 6, 5 mm: ***P = 0.0002, N = 6.

The distribution of Type I and Type II cells across the thickness of the distal IAS was determined by counting cell numbers exhibiting either Type I or Type II behaviour within the myenteric, middle or submucosal third of the muscle layer (Fig. 2A). Both cell types were present through the thickness of the muscle layer, however significantly greater numbers of Type II cells (63.7%) were observed than Type I cells (36.3%). Type II cells were greatest by more than a factor of two within the middle portion of circular muscle layer (*P* = 0.0001), which represents a novel distribution for pacemaker cells compared to other regions of the GI tract, i.e., ICC-MY and ICC-SM form networks along the margins of the circular muscle layer in other GI regions^11^. Although this study focused upon the distal end of the IAS, additional measurements were also undertaken to evaluate whether cell numbers within the muscle interior changed in the proximal direction (Fig. 2B). Type II cells declined orally, reaching undetectable levels 2–3 mm from the distal edge whereas Type I cells increased. These data add further support to our proposal that Type II cells represent a unique population of ICC-IM that is restricted to the IAS.

### Dependence of Ca^2+^ transients on extracellular Ca^2+^ and release of Ca^2+^ from stores

The dependence upon extracellular Ca^2+^ for the distinct behaviours of Type I and Type II cells was evaluated. Ca^2+^ transients were recorded from Type I and Type II cells before and during superfusion of Ca^2+^ free KRBS plus 0.5 mM EGTA. In Type I cells Ca^2+^ transients ceased 10.2 ± 0.6 min after beginning perfusion with Ca^2+^ free KRBS whereas Ca^2+^ transients in Type II cells were abolished after only 6.4 ± 0.7 min of Ca^2+^ free KRBS. These data indicate that both cell types are dependent to some extent on extracellular Ca^2+^ but that Type I cells are significantly more resistant to Ca^2+^ removal (*P* = 0.003; Supplemental Fig. S2). Additional experiments were undertaken to determine whether Ca^2+^ transients are due to Ca^2+^ release from the endoplasmic reticulum. The Ca^2+^-ATPase inhibitor CPA (10 µM) completely abolished Ca^2+^ transients in both cell types indicating their dependence on Ca^2+^ release from the endoplasmic reticulum (n = 12, N = 5, data not shown).

### Dependence of Ca^2+^ transients on Cav_L_

**Figure 3:**
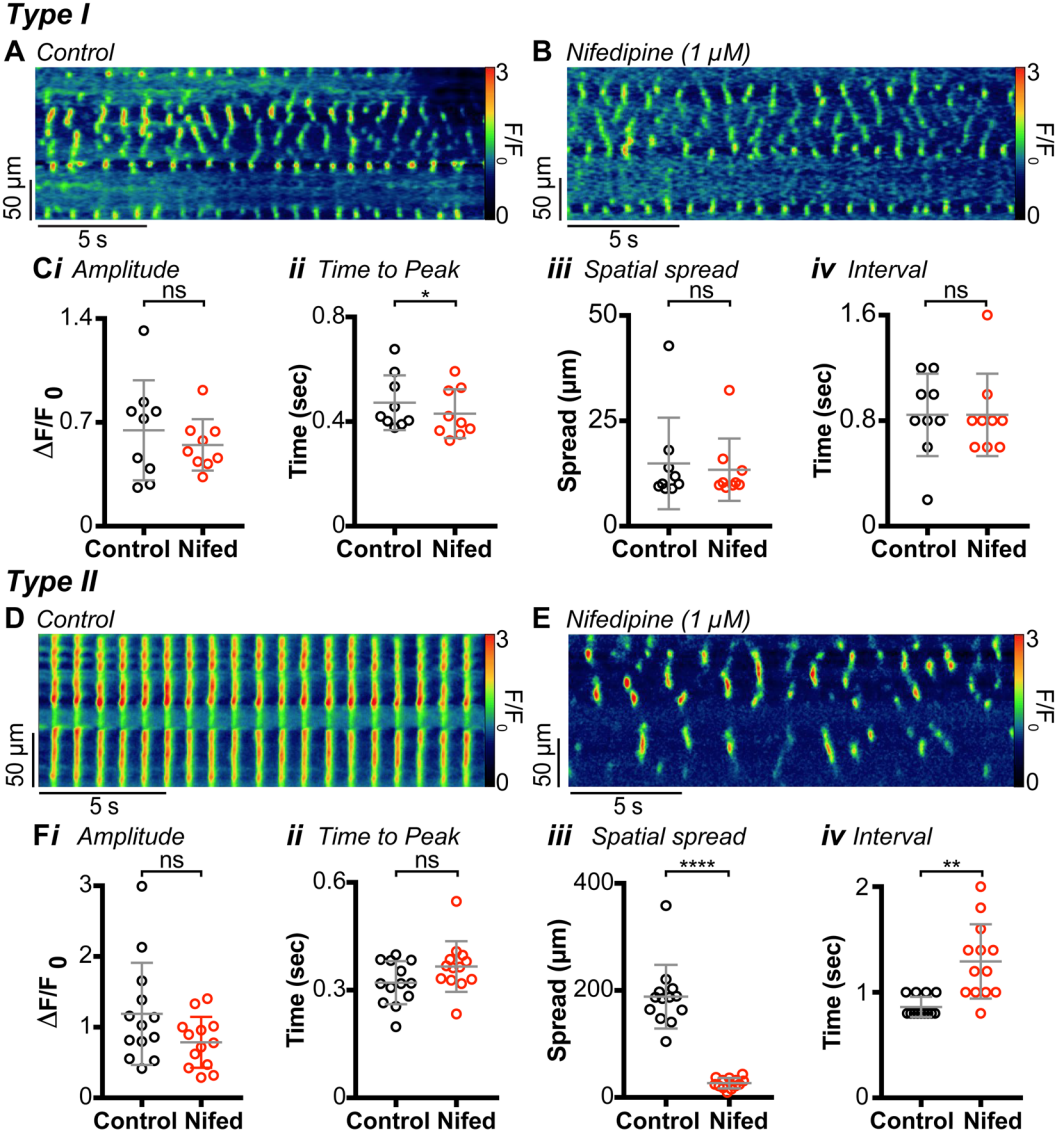
Ca^2+^ transients in Type I intramuscular interstitial cells of Cajal (ICC-IM) are not dependent on Ca^2+^ influx via Cav_L_, but rhythmic, global Ca^2+^ transients in Type II ICC-IM are. **(A)** Spatio-temporal (ST) map of a Type I ICC-IM under control conditions. **(B)** ST map of the same cell as **(A)** in the presence of 1 µM nifedipine. **(C)** Scatter plots summarising the effect of nifedipine (Nifed) on **(*i*)** amplitude (*P* = 0.2041), **(*ii*)** time to peak (*P* = 0.0146), **(*iii*)** spatial spread (*P* = 0.2793) and **(i*v*)** modal interval (*P* = 0.9999), in Type I cells (paired t test; n = 9, N = 4). **(D**,**E)** Representative ST maps of the same cell before **(D)** and after **(E)** application of 1 µM nifedipine. **(F)** Scatter plots summarising the effect of nifedipine on **(*i*)** amplitude (*P* = 0.1166), **(*ii*)** time to peak (*P* = 0.0912), **(*iii*)** spatial spread (*P* = 0.0001) and **(i*v*)** modal interval (*P* = 0.0013) in Type II cells. Control= ○, nifedipine = ○ (paired t test; n = 13, N = 6).

IAS-SWs and tone are dependent upon Cav_L_^7,8,13,14,26^. Thus, we compared the sensitivity of Ca^2+^ transients in Type I and Type II cells to blockade of Cav_L_. Ca^2+^ transients were recorded from Type I and Type II cells before and during superfusion with the Cav_L_ antagonist nifedipine (1 µM). Nifedipine caused a small but significant decrease in the time to peak (*P* = 0.015) of Ca^2+^ transients in Type I cells but did not significantly change the amplitude (*P* = 0.204), spatial spread (*P* = 0.279) or the mean modal interval (*P* = 0.999) between Ca^2+^ transients (Fig. 3A–C).

In contrast, the rhythmic, synchronised Ca^2+^ transients in Type II cells (Fig. 3D) were blocked by nifedipine, with activity similar to the Ca^2+^ transients in Type I cells persisting (Fig. 3E). Specifically, there was a significant decrease in the spatial spread (*P* = 0.0001) of Ca^2+^ transients and a significant increase in the mean modal interval (*P* = 0.001) between Ca^2+^ transients (Fig. 3F). These changes indicate a shift in behaviour of Type II cells to a more ‘Type I-like’ pattern when Cav_L_ are blocked and suggest that the rhythmic Ca^2+^ transients in Type II cells are organised into global, or propagated, Ca^2+^ transients when a mechanism for voltage-dependent Ca^2+^ entry is available. Similar observations occurred with ICC-MY, the pacemaker cells of the small intestine, although the voltage-dependent conductance required in those cells was Cav_T_^39^.

### Dependence of Ca^2+^ transients on membrane potential

**Figure 4:**
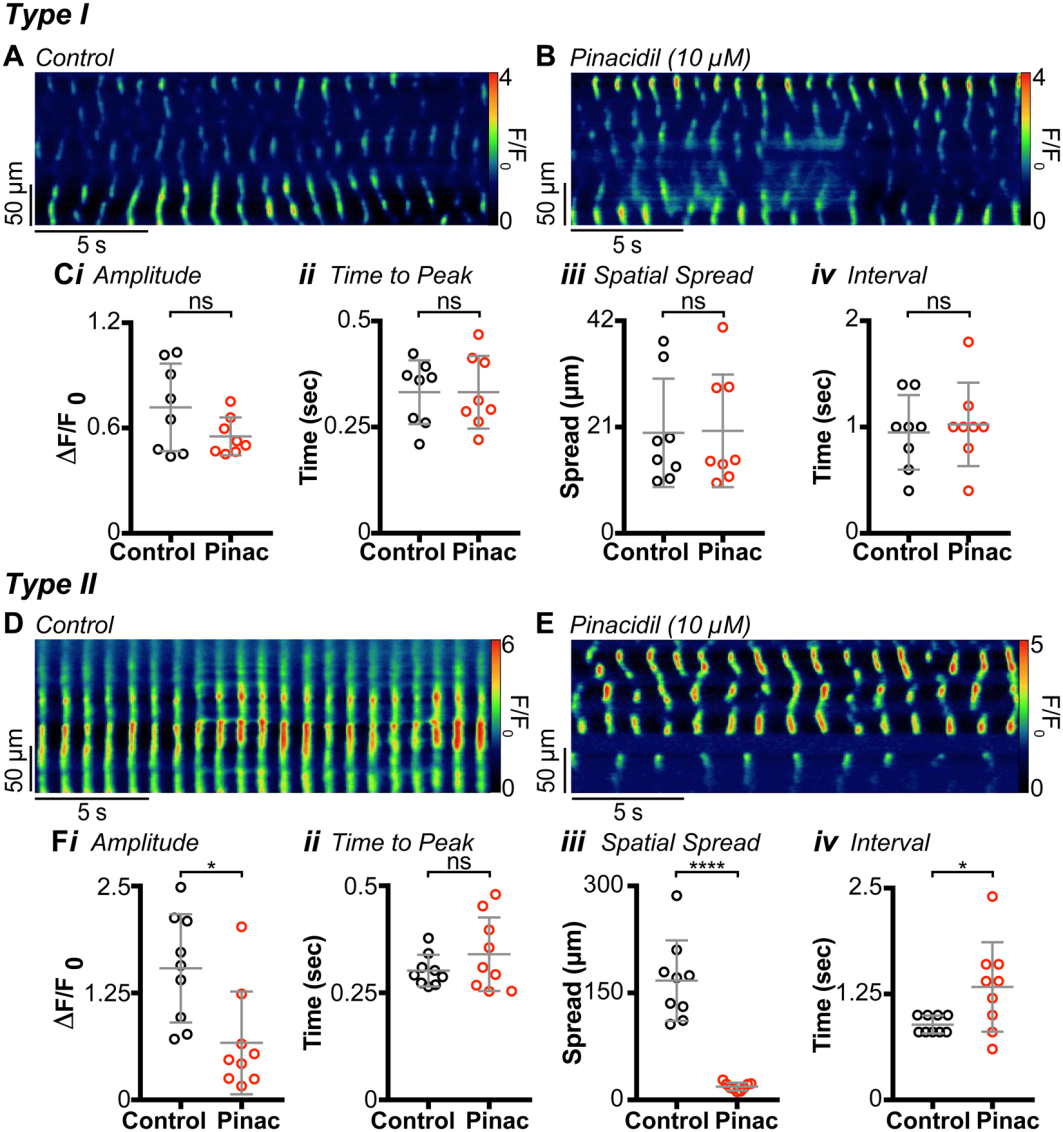
Type I cell Ca^2+^ transients are insensitive to changes in Em, whereas Type II cell Ca^2+^ transients are sensitive to changes in Em. **(A)** Spatio-temporal (ST) map of a Type I cell under control conditions. **(B)** ST map of the same cell as **(A)** in the presence of 10 µM pinacidil. **(C)** Scatter plots summarising the effect of pinacidil (Pinac) on **(*i*)** amplitude (*P* = 0.1245), **(*ii*)** time to peak (*P* = 0.9969), **(*iii*)** spatial spread (*P* = 0.83) and **(i*v*)** modal interval (*P* = 0.7318), in Type I cells (paired t test; n = 8, N = 3). **(D)** ST map of a Type II cell under control conditions. **(E)** ST map of the same cell as **(D)** in the presence of 10 µM pinacidil. Type II cell rhythmic Ca^2+^ transients are suppressed by changes in Em. **(F)** Scatter plots summarising the effect of pinacidil on **(*i*)** amplitude (*P* = 0.0138), **(*ii*)** time to peak (*P* = 0.85), **(*iii*)** spatial spread (*P* = 0.0001) and **(i*v*)** modal interval (*P* = 0.0353) in Type II cells. Control= ○, pinacidil = ○ (Paired t test; n = 9, N = 6).

IAS-SWs are abolished by membrane hyperpolarisation^10^, whereas Ca^2+^ transients in ICC-IM of the mouse colon are not^40^. We investigated whether Ca^2+^ transients in Type I or Type II cells are modified by membrane hyperpolarisation with the K_ATP_ channel agonist, pinacidil (Fig. 4). This compound has been shown to hyperpolarise Em and decrease phasic contractions and tone in the IAS^10^. Pinacidil (10 µM) had no significant effect on any of the Ca^2+^ transient parameters measured in Type I cells in the IAS (Fig. 4C), but pinacidil blocked the global, rhythmic Ca^2+^ transients in Type II cells (Fig. 4D,E). The global Ca^2+^ transients characteristic of Type II cells were replaced by Ca^2+^ transients similar to those in Type I cells (Fig. 4E), and a significant decrease in amplitude (*P* = 0.014) and spatial spread (*P* = 0.0001) was noted (Fig. 4F). There was also a significant increase in the mean modal interval (*P* = 0.035) between Ca^2+^ transients (Fig. 4F). These data further support the conclusion that global, rhythmic Ca^2+^ transients in Type II cells require voltage-dependent Ca^2+^ entry.

### Dependence of Ca^2+^ transients on ANO1

**Figure 5:**
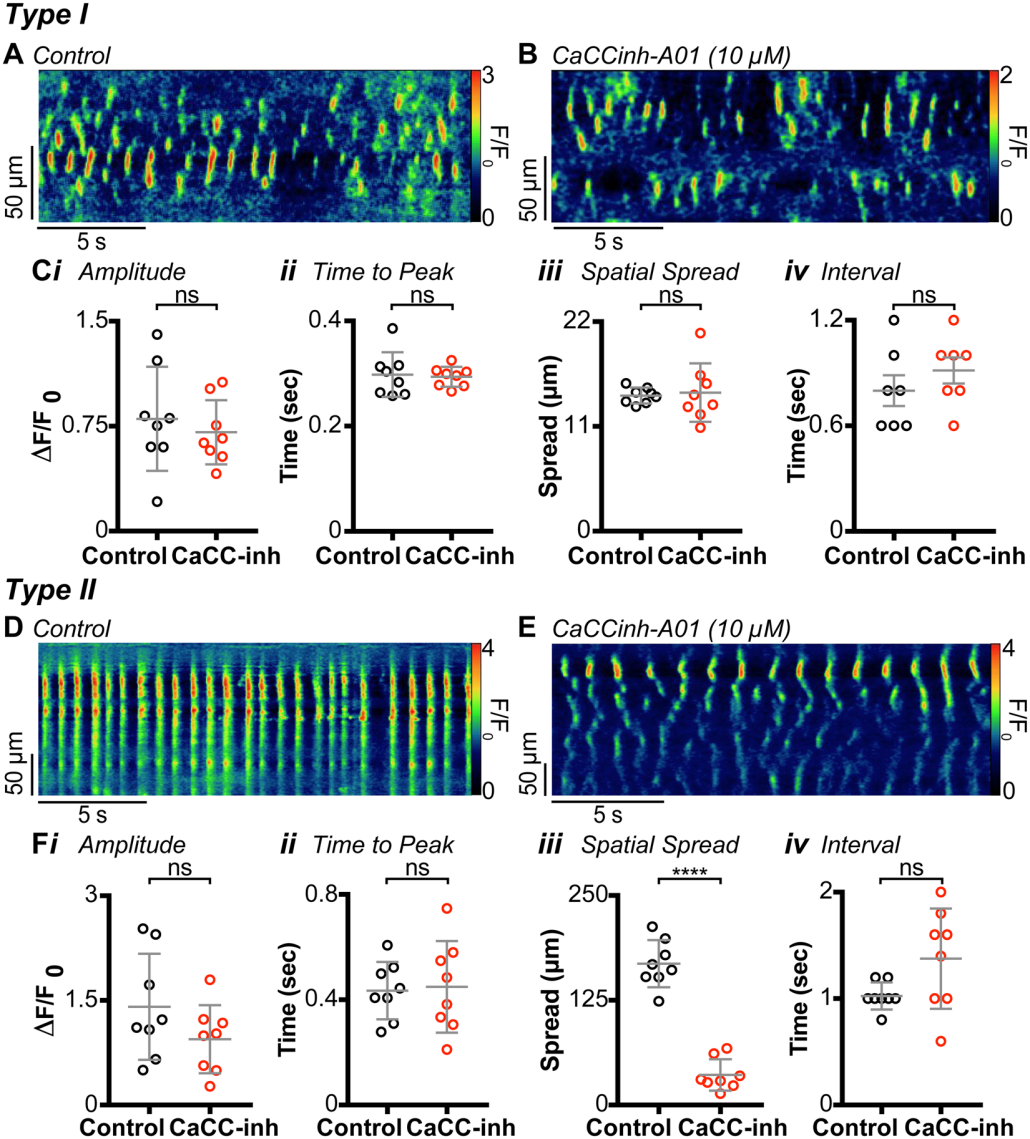
Inhibition of anoctamin 1 (ANO1) does not abolish Ca^2+^ transients in Type I cells, but it does abolish Type II rhythmic global cell Ca^2+^ transients. **(A)** Spatio-temporal (ST) map of Type I cell Ca^2+^ transients under control conditions. **(B)** ST map of Ca^2+^ activity in the same cell as **(A)** after the application of 10 µM CaCCinh-A01. **(C)** Scatter plots summarising the effect of CaCCinh-A01 (10 µM) on **(*i*)** amplitude (*P* = 0.46), **(*ii*)** time to peak (*P* = 0.7818), **(*iii*)** spatial spread (*P* = 0.8007) and **(i*v*)** modal interval (*P* = 0.103), in Type I cells (Paired t test; n = 8, N = 4). CaCCinh-A01 inhibits rhythmic Ca^2+^ transients in Type II cells. **(D)** ST map from a Type II cell under control conditions. **(E)** ST map from the same cell after the application of CaCCinh-A01 (10 µM). **(F)** Scatter plots summarising the effect of CaCCinh-A01 on **(*i*)** amplitude (*P* = 0.2191), **(*ii*)** time to peak (*P* = 0.7768), **(*iii*)** spatial spread (P = 0.0001) and **(i*v*)** modal interval (*P* = 0.0639) in Type II cells. Control= ○, CaCCinh-A01 = ○. (paired t test; n = 8, N = 6).

ANO1 plays a pivotal role in the generation of SWs in the GI tract including IAS-SWs^14^. For this reason, we examined the actions of the ANO1 blocker CaCCinh-A01 (10 µM) on Type I and Type II cells. Previously we have shown that 10 µM CaCCinh-A01 reduces spontaneous contractions in the mouse IAS by 83% while the contraction elicited by raising [K^+^]_o_ to 66 mM is reduced by only 14%^14^. Since the K^+^-induced contraction is entirely abolished by nifedipine but is largely intact following CaCCinh-A01, this concentration of CaCCinh-A01 appears to have limited direct effects upon Cav_L_. CaCCinh-A01 (10 µM) had no effect on Type I cells (Fig. 5A–C), but this compound shifted Ca^2+^ activity in Type II cells from rhythmic, global Ca^2+^ transients (Fig. 5D) to asynchronous Ca^2+^ transients similar to those observed in Type I cells (Fig. 5E). A significant decrease in the spatial spread of Ca^2+^ transients was also noted after exposure to CaCCinh-A01 (*P* = 0.0001; Fig. 5F). These data indicate that rhythmic, global Ca^2+^ transients in Type II cells depend on the availability of ANO1 channels.

### Immunohistochemical examination of the Kit-Cre-GCaMP6f mouse IAS

**Figure 6:**
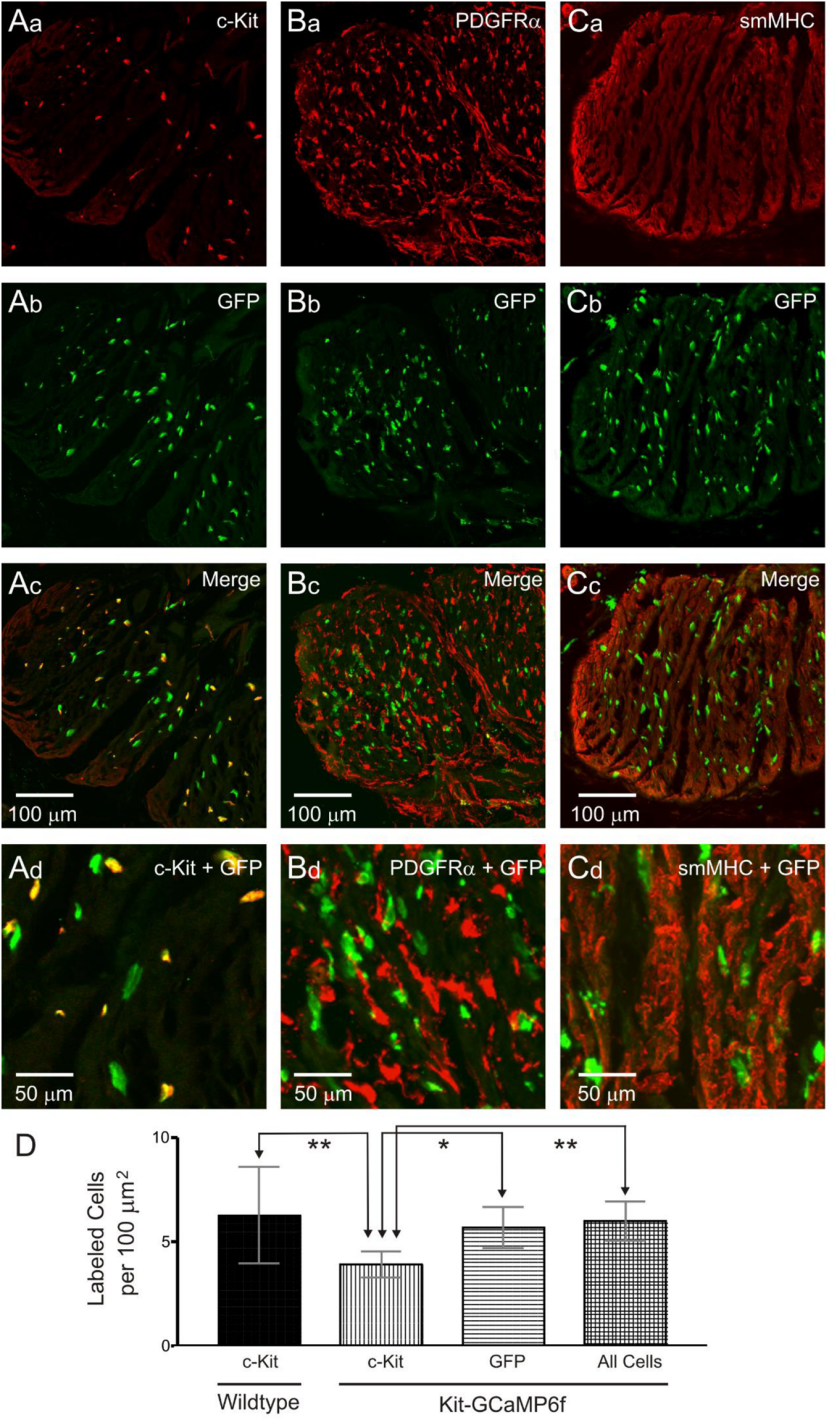
Immunohistochemical characterisation of GCaMP6f cells in the Kit-Cre-GCaMP6f mouse IAS. **(A–C)** Dual labelling images of GCaMP6f cells in the internal anal sphincter (IAS) labelled with an anti-GFP antibody in combination with either c-Kit, platelet-derived growth factor receptor alpha (PDGFRα) or smooth muscle myosin heavy chain (smMHC) antibodies. Higher magnification images are shown in the bottom panels (Ad, Bd, Cd). GFP^+^ cells were neither PDGFRα^+^ **(B)** nor smMHC^+^ **(C)** but the majority (61.6 ± 2.6%, N = 11) were c-Kit^+^ **(A)**. **(D)** Comparison of c-Kit^+^ cell density in *wildtype* mice (first bar) versus the density of c-Kit^+^ cells (second bar), GFP^+^ cells (third bar) and ‘all cells’ which includes all GFP^+^ cells plus cells that were c-Kit^+^/GFP^−^ (fourth bar) in Kit-Cre-GCaMP6f mice. The density of c-Kit^+^ cells in wildtype mice (N = 11) was significantly greater than in Kit-Cre-GCaMP6f mice (**P = 0.001) while wildtype c-Kit^+^ cell density was not different from the density of GFP^+^ cells (*P* = 0.727) or ‘all cells’ (*P* = 0.957) in Kit-Cre-GCaMP6f mice. Comparison of cell densities in Kit-Cre-GCaMP6f mice indicated that c-Kit^+^ cell density was significantly less than the densities of GFP^+^ cells (*P = 0.023) and ‘all cells’ (**P = 0.006). One-way ANOVA, *post-hoc* Tukey’s, N = 11.

We tested whether GCaMP6f is expressed selectively in ICC using dual labelling immunohistochemistry. In the GI tract, SMCs, ICC and PDGFRα^+^ cells form an electrical syncytium known as the “SIP” syncytium^11^. Therefore, we also examined expression in the other two cell types, i.e., SMCs and PDGFRα^+^ cells. Selective labelling of ICC, PDGFRα^+^ cells and SMCs was achieved using antibodies for c-Kit, PDGFRα and smMHC, respectively, while GCaMP6f^+^ cells were labelled with an antibody for GFP. GFP labelling did not overlap with either PDGFRα (Fig. 6B) or smMHC (Fig. 6C) indicating that GFP^+^ cells were neither PDGFRα^+^ cells nor SMCs. In contrast, the majority of labelled cells (60.4 ± 2.4%, n = 11) were GFP^+^/c-Kit^+^ while 34% were GFP^+^/c-Kit^−^ (34.2 ± 2.7%, n = 11; Fig. 6A). A final small percentage was GFP^−^/c-Kit^+^ (5.4 ± 1.0%, n = 11). We reasoned that the reduced expression of c-Kit in GFP^+^ cells may occur as a result of the methodology used to achieve specific GCaMP6f expression in ICC, i.e., reduction of Kit alleles from two to one (see methods). Therefore, we compared the density of c-Kit^+^ cells in wildtype and Kit-Cre-GCaMP6f mice (Fig. 6D). The cell density of c-Kit^+^ cells in Kit-Cre-GCaMP6f mice was found to be significantly less (*P* = 0.001) than that of wildtype mice whereas GFP^+^ density in Kit-Cre-GCaMP6f mice was similar (*P* = 0.957) to c-Kit density in wildtype mice. In summary, (1) GCaMP6f expression is coupled to c-Kit, (2) GCaMP6f cells are neither SMCs nor PDGFRα^+^ cells, and (3) GCaMP6f cells have a similar distribution and density as c-Kit^+^ cells in wildtype mice. These data suggest that GCaMP6f cells are ICC-IM even though c-Kit expression in some cells is low.

## Discussion

The IAS generates SWs that initiate phasic contractions which can summate to produce tone^10^. The present study examined the possible link between ICC-IM and pacemaker activity (i.e., SWs) in the IAS. To this end, Cre-lox methodologies were used to generate mice containing the genetically encoded Ca^2+^ indicator GCaMP6f in ICC (Kit-Cre-GCaMP6f). With the aid of these mice Ca^2+^ transients were examined in ICC-IM *in situ* within the IAS. An interesting outcome of this study was that two distinct sub-populations of cells were present. The first population (Type I ICC-IM) exhibited asynchronous Ca^2+^ transients similar to the ICC-IM that are involved in neuromuscular transmission in other GI muscles^11,16,18,40–48^. The second population (Type II ICC-IM) was novel and displayed spatiotemporal and pharmacological properties consistent with these cells generating pacemaker activity in the IAS.

### GCaMP6f is expressed in c-Kit^+^ spindle-shaped cells of the IAS

Immunohistochemical studies of the mouse rectoanal region have shown that ICC-MY and ICC-SM populations decline aborally reaching undetectable levels at the distal IAS whereas spindle-shaped ICC-IM are found throughout^23^. The present study supports this assessment since GCaMP6f expression in the distal IAS was limited to spindle-shaped intramuscular cells. Immunohistochemical examination of Kit-Cre-GCaMP6f tissues revealed that GCaMP6f (GFP^+^) cells. were smMHC^−^/PDGFRα^−^ indicating they were neither SMCs nor PDGFRα^+^ cells. As described in Methods, the Kit-Cre^ERT2^ mice used to produce Kit-Cre-GCaMP6f mice were generated by inserting Cre^ERT2^ in exon 1 of the Kit gene on a single allele^46^. Since, Cre^ERT2^ is expressed in place of *Kit* on one allele, Kit-Cre-GCaMP6f mice may have reduced *Kit* gene expression. None-the-less, the majority of GCaMP6f cells were c-Kit^+^ and the density of GCaMP6f cells in Kit-Cre-GCaMP6f mice was equivalent to the density of c-Kit^+^ cells in wildtype mice. Taken together, these data suggest that GCaMP6f expressing cells are ICC-IM.

### Two distinct populations of GCaMP6f cells are present in the IAS

Ca^2+^ imaging of GCaMP6f cells in the distal IAS revealed two distinct functional populations, i.e., Type I and Type II cells. Type I cells exhibited localised Ca^2+^ transients originating from multiple active sites each spreading a short distance. These Ca^2+^ transients were asynchronous both within and between neighbouring cells. Ca^2+^ transients in Type I cells were also smaller in amplitude and reached peak intensity faster than Ca^2+^ transients in Type II cells. The asynchronous activity described here for Type I cells is similar to that previously described for ICC-IM in the mouse colon and small intestine^40,47^. A large body of evidence from other GI muscles suggests that ICC-IM (and ICC-DMP in small intestine) serve as post-junctional targets for excitatory cholinergic and inhibitory nitrergic neuromuscular transmission^11,16,18,41–48^. It is likely that the Type I cells described here serve a similar role in the IAS. Indeed, functional nitrergic and cholinergic pathways are present in the mouse IAS^7,13^ and we have previously identified expression of guanylate cyclase and cGMP-dependent protein kinase in ICC-IM^49^. Ca^2+^ release events were independent of ANO1 but it is still possible that neurotransmitter-induced changes in Ca^2+^ release modify ANO1 activity giving rise to junction potentials (i.e., changes in Em) that change the contractile behaviour of adjacent SMCs.

In contrast, the activity of Type II cells consisted of rhythmic, global Ca^2+^ transients. Furthermore, the activity of neighbouring Type II cells was synchronised. In other GI regions, pacemaker ICC form networks at the myenteric or submucosal edges of the circular muscle, and SWs spread actively through these networks conducting to coupled SMCs as the wave of activity progresses^11^. In the IAS, synchronisation of Type II cells occurs in spite of the fact that these cells do not form a network with one another. However, ICC-IM are electrically coupled to SMCs via gap junctions^11,50,51^. Thus, SMCs may act as a bridge in the propagation of SW activity between ICC-IM. This is a novel model of SW propagation that may be unique to the IAS.

Type I and Type II cells were distributed across the thickness of the distal IAS but the changes occurring in their density in the oral direction differed. Type I cell density increased moderately in the proximal direction. In contrast, Type II cell density declined proximally reaching negligible levels 2 to 3 mm from the distal edge indicating that these cells are unique to the IAS. In like manner, previous functional studies have shown that the amplitude and frequency of SWs^23^ and SMC Ca^2+^ transients^52^ are greatest at the distal IAS and decline proximally. Thus, the anatomical location of Type II cells in the anorectum coincides with the functional distribution of SWs; an observation consistent with a role for Type II cells in the generation of SWs.

### Cav_L_ is critical for rhythmic Ca^2+^ events in Type II cells

Cav_L_ plays a critical role in the generation of SWs and tone in the IAS^8,12–14,26^. In a similar manner, rhythmic, global Ca^2+^ transients in Type II cells were abolished by blocking Cav_L_ with nifedipine or by hyperpolarisation of the Em with the K_ATP_ agonist pinacidil. In contrast, asynchronous, localised Ca^2+^ transients of Type I cells persisted following both procedures. Interestingly, following Cav_L_ blockade, the rhythmic, global Ca^2+^ transients observed in Type II cells were replaced by smaller amplitude asynchronous, localised Ca^2+^ transients similar in time course and amplitude to those of Type I cells indicating a common underlying feature of Type I and Type II cells.

As mentioned in the introduction, “resting” Em (i.e., the level of Em between SWs) is more depolarised in the IAS than intestine making Cav_L_ the primary mediator of SWs rather than Cav_T_^7,8,30^. The depolarised status of Em is likely the result of low resting K^+^ conductance (G_K_) since increasing G_K_ with pinacidil hyperpolarises Em by more than 20 mV, blocking SWs^10,53^ and global Ca^2+^ transients in ICC (present study). In like manner, purinergic neuromuscular transmission, which activates small conductance Ca^2+^-activated K^+^ channels on PDGFRα^+^ cells^54–57^, also hyperpolarises Em in the mouse IAS by more than 20 mV, again abolishing SWs^7,26^. Although nerve stimulation can dramatically alter the electrical and contractile activity of the *in vitro* mouse IAS^7,13,26,49,58^, blockers of cholinergic, purinergic and nitrergic transmission as well as the neurotoxin TTX have little or no effect upon the basal electrical and contractile activity in the mouse IAS^7,14,49,58^. Hence, the properties of Ca^2+^ transients described in this study are intrinsic to the Type I and Type II ICC-IM examined rather than to neural inputs.

### Relationship of Type I and Type II cell Ca^2+^ events to Ca^2+^ release and ANO1

Substantial evidence indicates that SW generation in ICC begins with localised release of Ca^2+^ from stores followed by activation of ANO1, depolarisation, activation of voltage-gated Ca^2+^ channels and finally a fully developed SW^36,59–61^. To examine the role of Ca^2+^ release in Type I and Type II cells, extracellular Ca^2+^ was removed or the Ca^2+^-ATPase inhibitor CPA was added. Ca^2+^ removal abolished all Ca^2+^ transients in Type I and Type II cells although the time required to produce full inhibition was greater for Type I cells (10.2 min) than Type II cells (6.4 min). This difference in time course may reflect the greater ongoing entry of Ca^2+^ into Type II cells via Cav_L_ and/or difference(s) in the mechanism(s) contributing to the uptake and storage of Ca^2+^ by the endoplasmic reticulum (ER). Asynchronous Ca^2+^ transients in Type I and Type II cells were also abolished by addition of the SERCA pump inhibitor CPA providing evidence they are due to Ca^2+^ release from the ER. The characteristics observed for asynchronous Ca^2+^ transients in Type I and Type II cells in the IAS are similar to those described for ICC-IM and ICC-DMP in the mouse colon and small intestine^40,47^. Furthermore, both studies conclude that Ca^2+^ release mechanisms involve inositol triphosphate receptors (InsP3Rs) as well as ryanodine receptors (RyRs) with ongoing refilling of the endoplasmic reticulum via Ca^2+^-ATPase.

**Figure 7:**
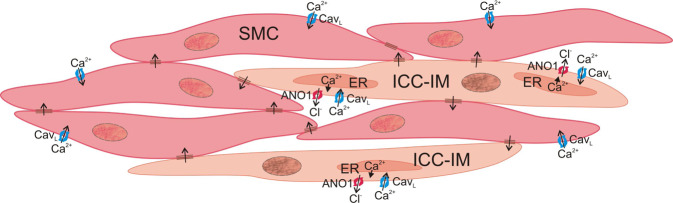
Proposed mechanism for the generation of pacemaker activity by Type II intramuscular interstitial cells of Cajal (Type II ICC-IM). In Type II ICC-IM, Ca^2+^ release from the endoplasmic reticulum activates the Ca^2+^-activated Cl^−^ channel ANO1 causing Cl^−^ efflux and membrane depolarisation. This in turn activates L-type voltage-dependent Ca^2+^ channels (Cav_L_) on ICC-IM resulting in Ca^2+^ influx and further activation of ANO1 which in turn leads to slow wave (SW) generation (observed as a rhythmic, global Ca^2+^ transient). Depolarisation is conducted to adjacent smooth muscle cells (SMCs) via gap junctions leading to Ca^2+^ influx in SMCs via Cav_L_ and thus, contraction. SWs, rhythmic, global Ca^2+^ transients and tone are all abolished by the ANO1 blocker CaCCinh-A01 (10 µM) and the Cav_L_ blocker nifedipine (1 µM) indicating that Type II ICC-IM represent a viable candidate for generating pacemaker activity in the IAS and that these cells may therefore play an important role in tone generation. This cartoon was drawn using CorelDraw 2019 Software.

Blockade of ANO1 with CaCCinh-A01 had similar effects on Type I and Type II cells to those observed with nifedipine or pinacidil. CaCCinh-A01 failed to block asynchronous localised Ca^2+^ transients in Type I cells while rhythmic, global Ca^2+^ transients in Type II cells were replaced by asynchronous localised Ca^2+^ transients. These data indicate that Cav_L_, Ca^2+^ release and ANO1 each contribute to the generation of synchronised rhythmic Ca^2+^ transients in Type II cells; observations consistent with SW generation in ICC (see Fig. 7). The very minimal effect of ANO1 inhibition on Ca^2+^ transients in Type I cells was interesting since there is evidence that spontaneous transient inward currents (STICs) initiated by ANO1 in ICC-IM contribute to regulating resting Em and modulation of Em by nerves^62,63^. However, since nifedipine, pinacidil and CaCCinh-A01 all have minimal effects on Ca^2+^ transients in Type I cells it supports the general consensus that Ca^2+^ transients in ICC-IM (other than Type II cells in the IAS) are voltage-independent events.

### Relationship of SWs to tone development in the IAS

The IAS differs from the majority of GI muscles in that it generates tone to raise pressure in the anal canal thereby facilitating faecal continence^1,2,10^. Cav_L_ channels play a fundamental role in this process since tone is blocked by manoeuvres which inhibit Cav_L_ activity^12–14,64–66^. A common feature of the IAS in all animal species studied is SWs^6–10^. Like tone, SWs are abolished by inhibitors of Cav_L_ activity^8,9,14,26,67^ indicating an important link between SWs and tone. One possible way in which phasic SWs can give rise to tone is via a partial (incomplete) tetanus type mechanism like that first described for skeletal muscle. Partial tetanus occurs as stimulus frequency is increased beyond a certain level, leading to an inability of Ca^2+^ removal to keep up with Ca^2+^ delivery and thus resulting in increased basal cytoplasmic [Ca^2+^]^68^. This “partial tetanus” consists of phasic contractions superimposed upon tone. SW frequency in the mouse IAS is quite high (i.e., around 70 cpm) and we have recently provided evidence for a partial tetanus type mechanism in studies of the SM-GCaMP mouse IAS^52^. In larger animal species (e.g., dog, monkey) the IAS is composed of numerous muscle bundles separated by wide connective tissue septa^19,21^. The degree to which these muscle bundles are electrically coupled is still unclear. Thus, an additional mechanism by which SWs may give rise to tone in larger animals is via summation of asynchronous phasic events arising from poorly coupled muscle bundles. In like manner, skeletal postural muscles can maintain tone through the asynchronous firing of motor units^69^. Finally, the overall depolarised level of Em in the IAS likely results in some Ca^2+^ entry via Cav_L_ that is time-independent (i.e., window current)^10^.

In summary, Ca^2+^ transients in ICC-IM were imaged *in situ* in Kit-Cre-GCaMP6f mice to examine the possibility that these cells are responsible for SW generation in the IAS. Two distinct sub-populations of ICC-IM were identified. The first, Type I ICC-IM, exhibited asynchronous Ca^2+^ transients with properties resembling those of ICC-IM in other GI muscles that participate in enteric neuromuscular transmission. The second, Type II ICC-IM, exhibited Ca^2+^ transients with spatiotemporal and pharmacological properties novel for ICC-IM but very consistent with the behaviour of pacemaker ICC. Type II ICC-IM exhibited rhythmic, global Ca^2+^ transients that were synchronised with adjacent Type II cells. These events were dependent upon Cav_L_ as well as ANO1; a Ca^2+^-activated Cl^−^ channel that is highly expressed in ICC-IM of the mouse IAS^14^. From these data we conclude that Type II ICC-IM are a viable candidate for the generation of pacemaker activity in the mouse IAS. Since tone in the IAS is also dependent upon Cav_L_ and ANO1^14^, it follows that Type II ICC-IM may also play an important physiological role in the regulation of anal pressure and maintenance of faecal continence.

## Methods

### Animals

In order to study intracellular Ca^2+^ activity in ICC, the genetically encoded Ca^2+^ indicator GCaMP6f was expressed specifically in ICC. GCaMP6f-lox^+^ mice (B6;129S-*Gt(ROSA)*^*26S*^*°*^*rtm95*.*1(CAG-GCaMP6f)Hze*^/J; The Jackson Laboratory; strain number: 024105) were crossed with Kit^+/Cre-ERT2^ mice (gift from Dr. D. Saur; Technical University of Munich, Munich, Germany) to generate Kit-Cre-GCaMP6f mice. As previously described, Kit-Cre^ERT2^ mice were generated by inserting Cre^ERT2^ into exon 1 of the Kit gene on a single allele^46^ thus confining Kit expression to one allele. To activate Cre-recombinase and induce GCaMP6f expression, mice were injected with tamoxifen at 6–8 weeks old (2 mg for three consecutive days) as previously described^47^. Animals were sacrificed 10–30 days after tamoxifen injection. For cell density studies, wildtype C57Bl/6 mice (6–8 weeks old, The Jackson Laboratory) were also used. Mice were euthanised with isoflurane (Baxter) inhalation and cervical dislocation. All procedures were approved by the Institutional Animal Use and Care Committee at the University of Nevada, Reno. Animals used and experiments performed were also in accordance with the National Institutes of Health Guide for the Care and Use of Laboratory Animals.

### Tissue preparation

Rectoanal tissues were removed and placed in a Sylgard-lined dissection dish with ice cold Krebs-Ringer bicarbonate solution (KRBS) of the following composition (in mM): 118 NaCl, 4.7 KCl, 2.5 CaCl_2_, 23.8 NaHCO_3_, 1.2 KH_2_PO_4_, 11 dextrose. KRBS had a pH of 7.4 after bubbling to equilibrium with 95% O_2_/5% CO_2._ Flat sheet preparations of the rectoanal region were prepared for Ca^2+^ imaging experiments by clearing the tissue of surrounding skeletal muscle including the external anal sphincter and fat, cutting the tissue open from rectum to anus and removing the mucosal layer to expose the inner circular layer of the muscularis externa. Tissues were pinned flat, with the circular muscle facing upwards in a 60 mm Sylgard/charcoal-lined dish. To rule out the possible excitatory effects of cholinergic motor neurons all experiments were carried out in the presence of atropine (1 µM).

### Calcium imaging

Tissues were continually perfused with oxygenated KRBS at 37 °C and left to equilibrate for 1 hour. For experiments determining the reliance of each cell type on extracellular Ca^2+^ the tissue was perfused with Ca^2+^ free KRBS plus 0.5 mM EGTA. Images were obtained with an upright confocal microscope (Olympus) equipped with a spinning disc (CSU-X1, Yokogawa) and an Andor iXon EMCCD camera (Andor Technology, Belfast) to detect images. GCaMP6f was excited using a 488 laser (Coherent OBIS) and all recordings were acquired with a 20 × 1.00 NA XLUMPlanFL N lens (Olympus, Japan) at a frame rate of 30 frames per second. All recordings, unless otherwise indicated were made from GCaMP6f cells at the distal extremity of the IAS.

### Calcium imaging analysis

Recordings of Ca^2+^ activity in ICC-IM of the IAS were imported into custom analysis software (Volumetry G8d, provided by Dr. Grant W. Hennig). If necessary, recordings were motion-stabilised to ensure accurate Ca^2+^ transient analysis. De-bleaching techniques were applied where appropriate in order to remove background fluorescence. Spatio-temporal (ST) maps were created for specific cells of interest by rotating image stacks so that ICC-IM were orientated vertically and drawing a region of interest (ROI) around the visible part of the cell. Further Ca^2+^ transient analysis was carried out by importing the ST maps into Image J (version 1.8, National Institutes of Health, MD, USA) as previously described^39,70,71^. Briefly, ST maps were normalised to obtain F/F_0_ by dividing the image by the mean intensity of the cell between Ca^2+^ transients. Active sites of Ca^2+^ activity, defined as sites that Ca^2+^ transients consistently originated from were analysed over a 10 s period (i.e. some cells had Ca^2+^ transients that originated from multiple active sites whereas others had Ca^2+^ transients that originated from only one active site, see descriptions of Type I and Type II cells in the results section). The amplitude (expressed as Δ F/F_0_), time to peak (time from trough to peak of a Ca^2+^ transient), spatial spread (distance Ca^2+^ transient spread along the cell, measured using the ‘wave speed’ command) and interval (time from one peak of a Ca^2+^ transient to the next, expressed as modal) were measured.

The cellular distribution within the circular muscle layer was calculated using confocal microscopy at 20x magnification (400 µm × 400 µm) and focusing upon either the myenteric, middle, or submucosal third of the circular muscle layer. All cells in focus within that region were then assigned according to established behaviours (see further description in the results section).

### Immunohistochemistry

#### Specimen preparation and labelling

The distal GI tract was kept in a tube and the mucosa removed as described previously^23^. Tissues were fixed in ice-cold Zamboni’s fixative (2% paraformaldehyde) for 20 min at 20 °C before washing in 0.1 M PBS, dehydrated in graded sucrose solutions (5–20%), and frozen as previously described^22^. Frozen tissues were sectioned perpendicular to the circular muscle layer at a thickness of 10–12 µm with a Leica CM 3050 cryostat (Leica Microsystems, Wetzlar, Germany) and labelled with primary and secondary antibodies as previously described^14^. Sections were blocked using 1% bovine serum albumin (BSA) for 1 hr at 20 °C before incubation in the first primary antibody (anti-GFP, Abcam, Cambridge, MA, USA; 1:1000 dilution in Triton-X working solution) for 16 hours at 4 °C. Sections were washed with 0.1 M PBS before incubation in secondary antibody (Alexa Fluor anti-chicken 488, Invitrogen, Carlsbad, CA, USA; 1:1000 dilution). Subsequently, sections were washed in 0.1 M PBS prior to incubation with the second primary antibody (i.e., anti-PDGFRα, R&D Systems, Minneapolis, MN, USA; 1:1000 dilution, mSCFR, anti-c-Kit antibody, R&D Systems, Minneapolis, MN, USA; 1:1000 dilution or anti-smooth muscle myosin heavy chain (smMHC), Biomedical Technologies Inc., Stoughton, MA, USA; 1:100 dilution). Following incubation with the second primary antibody, sections were washed and incubated with secondary antibody (Alexa Fluor anti-goat 594 (PDGFRα, c-Kit) or Alexa Fluor anti-rabbit 594 (smMHC), Invitrogen, Carlsbad, CA, USA; 1:1000 dilution) as described above. After washing with 0.1 M PBS, slides were covered with coverslips using Aquamount mounting medium (Lerner Laboratories, Pittsburgh, PA). Wildtype mouse rectoanal tissues were dissected, fixed and labelled as described above. In this case, preparations were labelled with anti-c-Kit antibody (mSCFR) followed by Alexa Fluor anti-goat 594.

### Imaging of Immunohistochemical Labelling and Determination of Cell Density

Confocal images of tissue sections were imaged using a Zeiss LSM 510 Meta confocal microscope (Carl Zeiss, Thornwood, NY, USA). Images shown in figures are composites of Z-series stacks taken through a depth of 1–13 µm. Figures were prepared using Zeiss LSM 510 Images Examiner Software, Adobe Photoshop CS5 Software and CorelDraw 2019 Software.

Cell density was determined from cross sections of the distal IAS circular muscle layer of wildtype (C57Bl/6) and Kit-Cre-GCaMP6f mice. A line was drawn around the edge of the circular muscle layer using CorelDraw 2019 Software and then all green, red and yellow cells were counted within this area. Total area was determined from images in Adobe Photoshop CS5 Software. Cell density was then expressed as number of cells per 100 µm^2^.

### Statistics

Data is expressed as the mean ± SD. Statistical analysis was carried out using GraphPad Prism (version 7.0). Student’s paired or unpaired two-tailed t-test was used to compare two groups, i.e., control conditions versus drug (paired) or various parameters in Type I versus Type II cells (unpaired). Two-way ANOVA with Šidák *post-hoc* test was used to compare Type I and Type II cell distribution in Ca^2+^ imaging studies. One-way ANOVA with Tukey’s *post-hoc* test was used in comparing c-Kit^+^ and GFP^+^ cell numbers in immunohistochemical studies. A *P*-value of <0.05 was considered statistically significant. n= number of cells, N = number of animals.

### Drugs

Nifedipine, pinacidil, CPA (cyclopiazonic acid), EGTA (ethylene glycol-bis(2-aminoethylether)-N,N,N′,N′-tetraacetic acid), atropine and tamoxifen were purchased from Sigma-Aldrich (Saint Louis, MO, USA). CaCCinh-A01 was purchased from Tocris Bioscience (Minneapolis, MN, USA). Stock concentrations were made by dissolving in de-ionized water (pinacidil), ethanol (nifedipine) or DMSO (CaCCinh-A01). Final concentrations were achieved by further dilution in KRBS. EGTA was dissolved to final concentration in KRBS. Tamoxifen was first dissolved in ethanol before addition of safflower oil to make a final concentration of 20 mg ml^−1^.

## Supplementary information

Supplementary Information.

Supplementary Information2.

## Data Availability

The datasets generated during and/or analysed during the current study are available from the corresponding author on reasonable request.
