# Supplementary material for: A novel intramuscular Interstitial Cell of Cajal is a candidate for generating pacemaker activity in the mouse internal anal sphincter
Source: Sci Rep. 2020 Jun 25;10:10378. doi: 10.1038/s41598-020-67142-y (PMC7316801; doi:10.1038/s41598-020-67142-y)
Supplement: Supplementary file 1 — Supplementary Information. [file 41598_2020_67142_MOESM1_ESM.docx]

**Supplementary Information**

**A novel intramuscular Interstitial Cell of Cajal is a candidate for generating pacemaker activity in the mouse internal anal sphincter**

Karen I. Hannigan, Aaron P. Bossey, Holly J.L. Foulkes, Bernard T. Drumm, Salah A. Baker, Sean M. Ward, Kenton M. Sanders, Kathleen D. Keef and Caroline A. Cobine

Department of Physiology and Cell Biology, University of Nevada, Reno School of Medicine, Reno, NV 89557 USA

***Corresponding Author:** Caroline A. Cobine, Ph.D.

Department of Physiology and Cell Biology,

University of Nevada, Reno School of Medicine,

1664 N. Virginia St., MS 352,

Reno, NV 89557

USA

Phone: 1-775-682-8840

Fax: 1-775-784-6903

E-mail: ccobine@med.unr.edu

*Supplemental Fig S1.avi*

**Supplemental Figure S1:** Video recording taken from the distal edge of the Kit-Cre-GCaMP6f mouse internal anal sphincter (IAS) at 20x. This video shows Ca^2+^ transients within Type I (* and green text) and Type II cells (* and yellow text) and was used to produce the still image, spatio-temporal (ST) maps (taken from highlighted cells) and Ca^2+^ traces shown in Fig 1A-E.


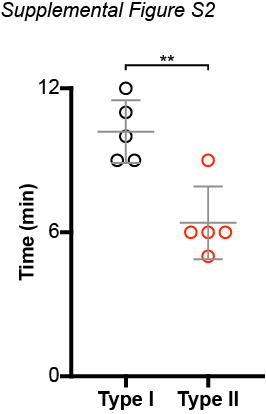


**Supplemental Figure S2:** Scatter plot summarising the time taken for Ca^2+^ transients in Type I (**o**) and Type II (**o**) cells to cease after superfusion with Ca^2+^ free KRBS plus 0.5 mM EGTA. Unpaired t-test, ***P*=0.003, N=5.
